# The Transcranial Light Therapy Improves Synaptic Plasticity in the Alzheimer’s Disease Mouse Model

**DOI:** 10.3390/brainsci12101272

**Published:** 2022-09-21

**Authors:** Débora Buendía, Tatiana Guncay, Macarena Oyanedel, Makarena Lemus, Alejandro Weinstein, Álvaro O. Ardiles, José Marcos, Adriana Fernandes, Renato Zângaro, Pablo Muñoz

**Affiliations:** 1 Programa de Engenharia Biomédica, Instituto de Engenharia Biomédica, Universidade Anhembi Morumbi—UAM, Rua Casa do Ator, 294, Sao Paulo 04546-001, Brazil; 2 Escuela de Ingeniería Civil Biomédica, Facultad de Ingeniería, Universidad de Valparaíso, General Cruz 222, Valparaíso 2362905, Chile; 3 Centro de Neurología Traslacional, Facultad de Medicina, Universidad de Valparaíso, Valparaíso 2341386, Chile; 4 Centro de Inovação, Tecnología e Educação—CITÉ, Parque Tecnológico de São José dos Campos, Estrada Dr. Altino Bondesan 500, São José dos Campos 12247-016, Brazil; 5 Centro Interdisciplinario de Neurociencia de Valparaíso, Facultad de Ciencias, Universidad de Valparaíso, Valparaíso 2360102, Chile; 6 Escuela de Medicina, Facultad de Medicina, Universidad de Valparaíso, Angamos 655, Viña del Mar 2540064, Chile; 7 Escuela de Ciencias Agrícolas y Veterinarias, Universidad Viña del Mar, Viña del Mar 2572007, Chile; 8 Centro de Investigaciones Biomédicas, Facultad de Medicina, Universidad de Valparaíso, Angamos 655, Viña del Mar 2540064, Chile

**Keywords:** Alzheimer’s disease, transcranial light therapy, LLLT, synaptic plasticity, cognitive functions, Alzheimer’s disease non-invasive treatment

## Abstract

Alzheimer’s disease (AD) is the main cause of dementia worldwide. Emerging non-invasive treatments such as photobiomodulation target the mitochondria to minimize brain damage, improving cognitive functions. In this work, an experimental design was carried out to evaluate the effect of transcranial light therapy (TLTC) on synaptic plasticity (SP) and cognitive functions in an AD animal model. Twenty-three mice were separated into two general groups: an APP/PS1 (ALZ) transgenic group and a wild-type (WT) group. Each group was randomly subdivided into two subgroups: mice with and without TLTC, depending on whether they would undergo treatment with TLTC. Cognitive function, measured through an object recognition task, showed non-significant improvement after TLTC. SP, on the other hand, was evaluated using four electrophysiological parameters from the Schaffer-CA1 collateral hippocampal synapses: excitatory field potentials (fEPSP), paired pulse facilitation (PPF), long-term depression (LTD), and long-term potentiation (LTP). An improvement was observed in subjects treated with TLTC, showing higher levels of LTP than those transgenic mice that were not exposed to the treatment. Therefore, the results obtained in this work showed that TLTC could be an efficient non-invasive treatment for AD-associated SP deficits.

## 1. Introduction

Alzheimer’s disease (AD) is the main cause of dementia worldwide [1]. This heterogeneous neurodegenerative disorder appears in 35% of individuals above 85+ and affects more than 5 million people in the United States [2], with an incidence and prevalence of 5% in Europe and 11.08 per 1000 person-years, respectively [3]. In Latin America, the prevalence of dementia has reached 7.1%, with AD being the most frequent type [4]. Conditions, such as age, female sex, diabetes, high blood pressure, physical inactivity, smoking, and mental inactivity, have been identified, along with genetic conditions, as the main risk factors [5,6,7,8,9].

The diagnosis of AD has changed over time, from purely pathological to clinical criteria, ending in a categorization based on biological biomarkers that evidence pathological changes in AD [10]. This evolution has allowed the development of a combined system that classifies the progression and severity of the disease through biomarkers and the degree of cognitive impairment, within what we now know as the AD continuum [11,12]. In this sense, both the decline in cognitive ability and the progression of AD biomarkers are part of a continuous process that begins before symptoms arise and develop over a long period [13,14], varying from cognitively normal, to mild cognitive impairment, or to dementia. Initial clinical symptoms are characterized by an early impairment in learning and memory, with later impairments observed in executive function, complex attention, language, visuospatial function, praxis, gnosis, and behavior [15]. Gaze, an automatic and reflexive process that involves eye movement, important for shared attention [16], is also affected in AD [17]. Since clinically observed cognitive symptoms are not always specific to AD [18], a flexible ATN classification system has been developed, including different biomarkers (fluid and imaging) of β-amyloid deposition (A), pathological tau (T), and neurodegeneration (N), where new biomarkers can be added to the three groups [19]. This ATN system supports the importance of amyloid β and tau as the characteristic definition of Alzheimer’s disease, recognizing that these markers may be present in many other pathologies [10].

Although the pathogenesis of the disease is not yet well defined, it has been defended that an imbalance in the proteostasis control mechanisms may be the trigger [20]. This alteration may result in the pathological accumulation of proteins in the brain, such as amyloid-beta peptide and Tau neurofibrillary tangles (NFTs), initializing a cascade of generalized neurotoxicity events, including oxidative stress, local inflammation, and hyper-phosphorylation, with an imbalance between various neurotransmitters, such as acetylcholine, dopamine, or serotonin. This may cause a deficit in synaptic transmission [21], leading to an impairment in synaptic plasticity (SP), which is the ability of neurons to modify the number, structure, and functions of synapses over time, including long-term potentiation (LTP) and long-term depression (LTD), among others [22]. Synaptic dysfunction has been mostly evidenced in the hippocampus and cortex, which are associated with the cognitive deficit observed in AD [23].

Based on the possible etiology, until 2020, the Food and Drug Administration (FDA) only approved four drugs for the treatment of AD [24]. These drugs are used to counterbalance the neurotransmitter imbalance of the disease: donepezil, galantamine and rivastigmine act as inhibitors of the acetylcholinesterase enzyme (AChE), and the memantine as a N-Methyl-D-aspartate (NMDA) receptor antagonist. However, these drugs do not prevent neural loss, brain atrophy and, consequently, the progressive cognitive decline observed in AD patients [25]. A new drug for AD has been approved based on a significant reduction of Aβ plaque in the brain, Aduhelm (aducanumab), but with significant controversy [26]. As a consequence, non-pharmacological alternatives are currently emerging and receiving increasing attention, such as the transcranial photobiomodulation (PBM), also known as low level light therapy (LLLT), which involves the delivery of red or near-infrared light into the brain (TLTC) [27]. The focus of this therapy is the mitochondrial oxidative stress that generates the environment for the production of NFTs and Aβ proteins. Different light sources have been used for PBM, such as lasers and light-emitting diodes (LED). Despite the controversy on whether the coherent monochromatic laser is superior to non-coherent LEDs [28], evidence suggests that LEDs perform equally well compared to lasers, with the advantages of being safe, presenting a lower cost, and a better suitability for home use [29].

Prior studies using both animal and human models have shown the beneficial effects of PBM in AD. In AD animal models, the most common effect is the reduction of amyloid plaques by a decrease of the Aβ peptide neuropathology [30,31,32,33]. In human models, PBM has shown beneficial neurocognitive effects in older adults while performing the psychomotor vigilance task, sustained attention, and memory task [34] Moreover, PBM treatment has improved cognition skills in a small sample of AD patients [35,36,37].

Although there is evidence showing that PBM increased SP in experimental cases of depressive disorders [38], stroke [39], and traumatic brain injury [40], there are few studies addressing neurodegenerative conditions such as AD. This is important, given that if PBM allows restoring excitatory synaptic transmission in the hippocampus of subjects with AD, and this, in turn, has a positive impact on the patient’s cognitive functions, PBM could be a good non-invasive treatment option for AD. Therefore, the main objective of this work is to evaluate, using an animal model, the ability of transcranial light therapy (TLTC) to increase SP and to improve cognitive functions in subjects with AD.

The main contribution of this research focuses on demonstrating how TLTC increases SP in subjects with AD, through the evaluation of electrophysiological parameters, being, to the best of our knowledge, one of the first works addressing this topic. The results provide further support to TLTC for its subsequent use as an alternative treatment for AD. In addition, a novel prototype and a new light therapy treatment protocol were established for the experimental design of this study.

## 2. Materials and Methods

### 2.1. Animals

C57BL/6 Wild type mice (WT group, Chicago, IL, USA) were used as control group and APP/PS1 transgenic mice, expressing the Swedish mutant for amyloid precursor protein (APP-SWE) and the exon 9 deletion mutant for presenilin (PSEN1 E9), obtained from Jackson Laboratory (Bar Harbor, ME, USA), were used as subjects with AD (ALZ group, Hervey Bay, QLD, Australia). Both groups were 9 months old and were housed at 22 ± 2 °C at constant humidity (55%) and with light/dark cycles of 12/12 h, with a light phase of 8:00 a.m. to 8:00 p.m. Food and water were provided ad libitum.

The experiment was carried out following the guidelines and regulations for the care and use of animals for scientific purposes registered in the ARRIVE standards for the Replacement, Refinement, and Reduction of Research Animals, and following the guidelines established by the Research Bioethics Committee of the Faculty of Pharmacy of the University of Valparaíso (CBI A 02/2021).

### 2.2. TLTC Device

The designed TLTC device complies with fundamental characteristics to achieve neuronal stimulation, such as: optical power less than 500 mW, power density between 1 and 5 W/cm^2^, electromagnetic spectrum between 600 and 1100 nm [41], and light penetration into the brain no greater than 3 cm. Specifically, our device uses a radiation source in the red range (visible) and with a peak at 630 nm, which according to Hamblin (2017) [42] is the most efficient wavelength for TLTC application, leading to low power consumption, small physical size, durability, and high reliability.

**Figure 1:**
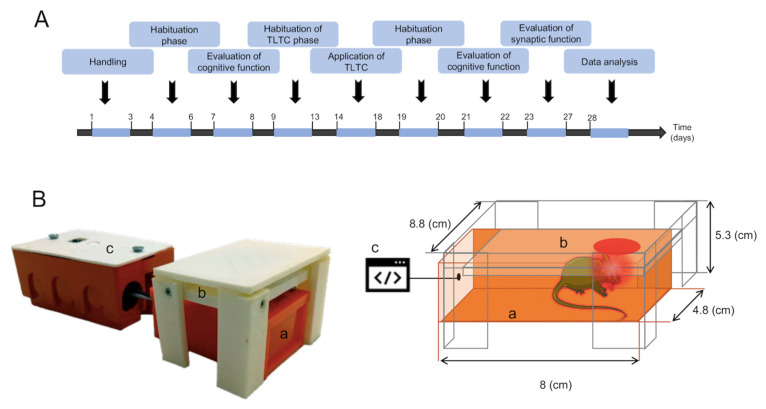
Scheme of the experimental design. (**A**) Timeline shows the sequence of events and behavioral testing of the animals. After handling and habituation, the animals It involves a stage of cognitive functions by recognizing new objects, applying light therapy, and analyzing synaptic plasticity using an experimental design for twenty-seven days. (**B**) Transcranial light therapy (TLTC) device. Left, Representative image of the TLTC device. Right, drawing of the TLTC device showing the three main components of the device: (a) box designed to contain the animal during the application of therapy, reducing handling and the stress factor, (b) platform where the light module is located, and (c) the control unit is the module programmed to deliver a constant light intensity for a period of time.

A prototype of the TLTC device was designed for the study, containing three main components: box, light module, and control unit, described as follows (Figure 1):

- Box: An acrylic box measuring 8 × 4.8 × 4.8 cm, with an opening at the top (2.3 cm^2^) was designed to reduce the stress factor in mice and to ensure transcranial irradiation and attenuation of light to other regions of the animal’s body.
- Light module: The module is based on the LSMCC-x4X3-LP single-color LED module. The module contains four high-power 5050SMD LEDs. Each LED has a viewing angle of 120 degrees, a wavelength of 630 nm, and 1 W of power. The coverage area of the module is 2.3 cm^2^. The module is powered by 12 volts and is controlled by a relay in the control unit. The light module is mounted on the top of the box.
- Control unit: The unit contains an electronic board with an ATmega328 microcontroller, a push button, a relay, and additional supporting components (voltage regulators, discrete transistors, connectors, etc.). When the push button is pressed, the microcontroller turns on the relay for 125 s, turning on the light module.

The TLTC device delivers a constant light intensity of 74.5 lx during the 125 s the light module is on. This corresponds to an energy equal to 100 J, an energy density of 43.5 J/cm^2^, and a power density of 0.35 W/cm^2^.

### 2.3. Experimental Design

Twenty-three 9-month old mice were used in the study. Mice were divided into two groups: WT (*n* = 12) and ALZ (*n* = 11). In turn, each group was randomly subdivided into two subgroups: control and experimental. Mice in the experimental group underwent TLTC, while mice in the control group did not. In summary, the four subgroups were WT (*n* = 5), WT + TLTC (*n* = 7), ALZ (*n* = 4), and ALZ + TLTC (*n* = 7). Next, the experimental design shown in Figure 1 was applied, starting with a handling phase, where the animals were manipulated by the experimenter for five minutes daily for three consecutive days. The habituation phase starts in day 4, placing mice in an open field arena consisting of a white, rectangular polyethylene box measuring 41 cm × 31 cm × 41 cm located in the same room where mice were kept while recording the animal’s activity. On the seventh and eighth days, the first evaluation of memory recognition was performed using the “Novel Object Recognition (NOR)” test. The task relies on an animal’s intrinsic preference for exploring novelty without additional external reinforcement, unlike many other behavioral tests involving a reinforcement with aversive and/or stressful events [43]. Mice in the experimental subgroups (WT + TLTC and ALZ + TLTC) underwent TLTC. On the ninth day, a new habituation phase was developed inside the cage, where the animals got used to entering the box of the TLTC device for 125 s. This habituation to the TLTC device was repeated for five consecutive days, after which TLTC was applied. During this time, mice enter the acrylic box to which they were accustomed previously (Figure 1(Ba)). However, this time, TLTC was applied to the animal’s head for 125 s for five consecutive days (day 14 to day 18). Once the TLTC was completed, a second cognitive evaluation was performed using the NOR test. Finally, SP was evaluated on day twenty (Figure 1A).

#### 2.3.1. Novel Object Recognition (NOR) Task

**Figure 2:**
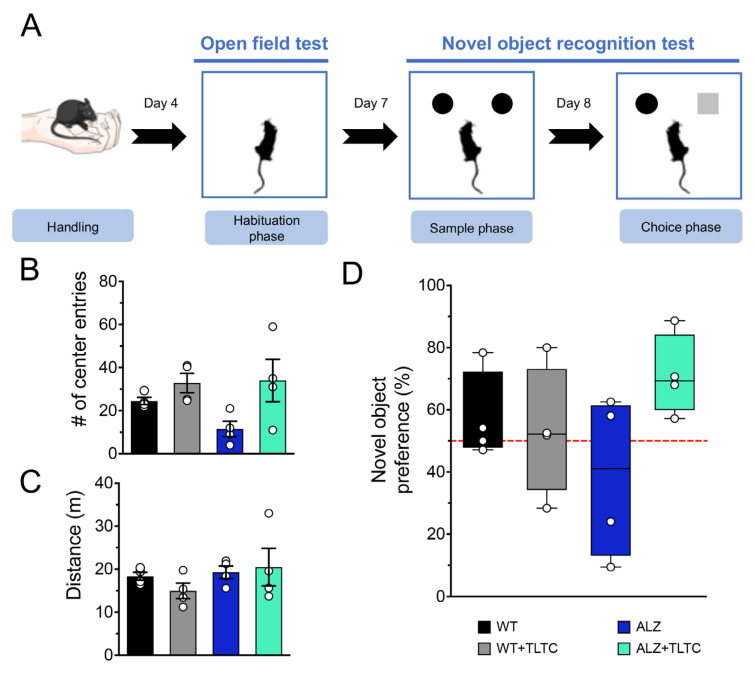
TLTC tends to improve novel object recognition memory in AD-bearing mice. (**A**) Diagram of the new object recognition procedure. The first phase is a stage of manipulation of the animals (handling), the second phase consists of the habituation to the experimental arena and open field test (OFT), the third phase consists of the object recognition phase (with two equal objects), and finally, the fourth phase is the choice phase, where one of the objects is replaced by a new object. (**B**) Number of times the mice entered the center of the experimental arena for transgenic mice with Alzheimer’s disease without (ALZ, blue bar) and with TLTC (ALZ + TLTC, green bar), and control group, also subdivided into mice without (WT, black bar) and with TLTC (WT + TLTC, gray bar). (**C**) Total distance traveled by the mice during the habituation phase. The OFT test shows no signs of anxiety in the mice, neither before nor after the TLTC was applied. (**D**) Percentage of the preference for the novel object. Animals from the ALZ + TLTC group showed a non-significant preference for the new object.

The NOR test included habituation, sample, and choice phases (Figure 2A). During the habituation phase, the animals freely explored the open field arena daily for five minutes for three consecutive days. To analyze the exploration time in the different areas of the arena, the ANY-MAZE version 4.99 program was used. The total exploration time inside the arena, in the periphery, and in the center was calculated.

The sample phase begins when the animals are exposed to two identical objects in texture, shape, and color, and are allowed to explore them freely for ten minutes (Figure 2A).

The exploration period is defined as the time during which the animal sniffs or touches the object with its front legs at a distance less than or equal to 1 cm. After 24 h following the last session of sample phase, the choice phase begins. In this phase the animal re-enters the behavioral arena, but one of the “familiar” objects is replaced by a “novel” object with a similar texture and size, but with a different shape. Each animal is given a 5-min period to explore both objects. It should be noted that in order to eliminate odors between the tests, the experimental apparatus and the objects in the arena were cleaned with 70% ethanol, after each test.

For the choice phase, the recognition index was calculated as the percentage of preference shown by the animal, which corresponds to the exploration time of the novel object (Tn) divided by the total exploration time, that is, the sum of the exploration times of the novel and the familiar object [44].

#### 2.3.2. Synaptic Plasticity

**Figure 3:**
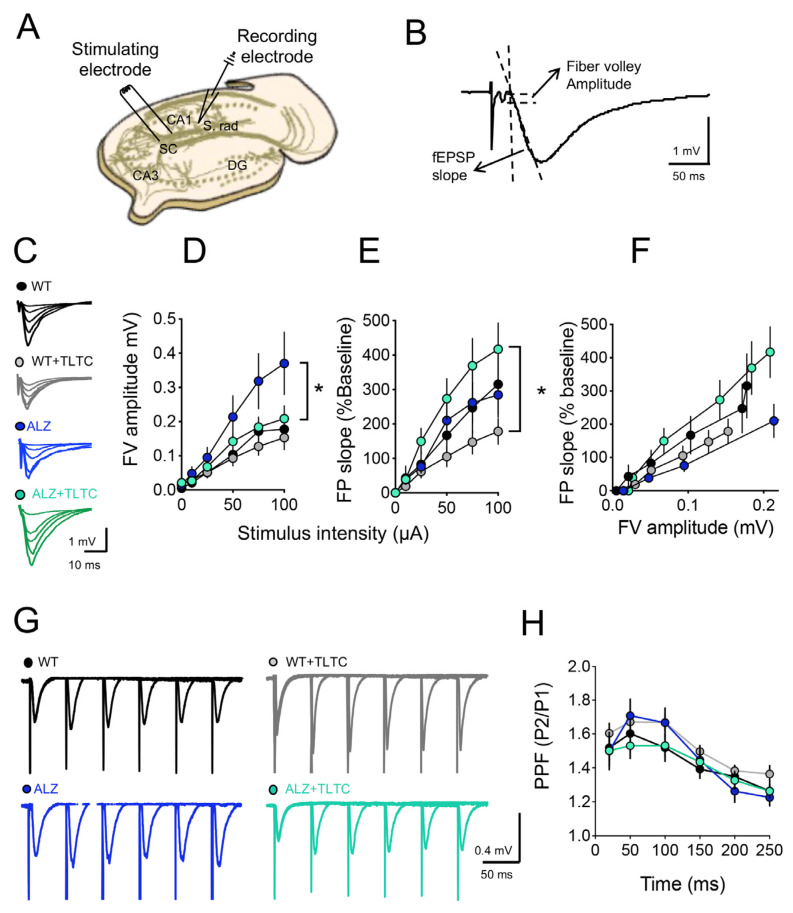
TLTC influences increased field excitatory postsynaptic potentials (fEPSP) and I/O signal in AD mice (ALZ). (**A**) A scheme of a cross-section of the hippocampus used in electrophysiology experiments. CA = Ammonis horn. DG = dentate gyrus. SC = Schaffer collaterals. S. radiatum = stratum radiatum. (**B**) Typical waveform of a fEPSP. Electrical stimulation of the SC causes a stimulus artifact, followed almost immediately by an increase in the presynaptic response, or fiber volley (FV). The amplitude of the VF is directly proportional to the amount of SC fibers activated by the stimulus. The slope of the fEPSP corresponds directly to the response obtained in the pyramidal CA1 neurons (postsynaptic) in response to the release of glutamate from the SC terminals. (**C**) Representative traces of fEPSPs recorded at different stimulus intensities for transgenic mice with Alzheimer’s disease without (ALZ, blue circles) and with TLTC (ALZ + TLTC, green circles), and a control group, also subdivided into mice without (WT, black circles) and with TLTC (WT + TLTC, gray circles). (**D**–**F**) Input-output curves (I/O) showing the relationship between the slope fEPSP (**D**) and the amplitude of the fiber volley (c) versus the intensity of the stimulus; and fiber volley amplitude and fEPSP slope (**F**). An increase in fEPSP slope was observed in ALZ + TLTC mice compared to WT + TLTC mice and an increase in fiber volley amplitude in ALZ mice compared to WT mice. (**G**) Representative traces showing paired fEPSPs responses at different time intervals (20–250 ms) for transgenic mice with Alzheimer’s disease without (ALZ, blue circles) and with TLTC (ALZ + TLTC, green circles), and the control group, also subdivided into mice without (WT, black circles) and with TLTC (WT + TLTC, gray circles). (**H**) The graph illustrates the paired-pulse facilitation (PPF) relationship versus the intervals between stimuli. * Indicates statistically significant difference.

For the electrophysiological experiments, mice were anesthetized and euthanized to extract their brains. Hippocampi were cut into 400 µm transversal slices using vibratome (VT 1200 S Leica Wtzlar, Alemania) and cold dissection buffer (in mM: 212.7 sucrose, 5KCL, 1 MgCl_2_, 2 CaCl_2_, 10 glucose, 1.25 NaH_2_PO_4_, 26 NaHCO_3_, pH 7.4 with 95% O_2_/5% CO_2_) [45]. One hour before recording, the hippocampal slices were stabilized and superfused with oxygenated artificial cerebrospinal fluid (ACSF) (in mM: 124 NaCl, 5 KCl, 1.25 NaH_2_PO_4_, 1 MgCl_2_, 2 CaCl_2_, 10 glucose, 26 NaHCO_3_, pH 7.4) bubbled with a mixture of 95% O_2_/5% CO_2_ [45]. Field excitatory postsynaptic potentials (fEPSP) were evoked by stimulating the Schaeffer collateral fibers with current square pulses of 0.2 ms, applied with concentric bipolar stimulation electrodes (FHC Inc., Bowdoinham, ME, USA), and recorded extracellularly with ACSF-filled glass microelectrodes (2–3 MΩ) in the stratum radiatum region of the CA1 area of the hippocampus (Figure 3A). Gradually increasing pulses from 25, 50, 75, 100, 150, to 200 microamps were applied to generate input/output (I/O) response curves using a constant current stimulator controlled by IGOR software. In this protocol, the input is represented by the maximum amplitude of the fiber volley (FV), and the output by the slope of the fEPSP (Figure 3B). Paired pulse facilitation (PPF) consists of evoking two consecutive stimuli with intervals between stimuli of 10, 20, 50, 100, 150 and 200 ms. For data analysis, the ratio of the second slope to the first is determined and plotted against the interval between stimuli. Finally, long-term synaptic plasticity experiments were performed after I/O and PPF determinations. For this, pulses were administered every 15 s that evoked half of the maximum response. Once the response stabilized, a response baseline was recorded for a minimum period of 20 min. To induce long-term depression (LTD), a low frequency stimulation (LFS) protocol consisting of 900 pulses at 1 Hz (one pulse every second for 15 min) was applied. Long-term potentiation (LTP) was evoked by theta-burst stimulation (TBS), which consists of four high-frequency tetanic stimulation trains applied at 0.1 Hz. Each train consists of ten tetanus (burst or bursts) separated by 200 ms (5 Hz) and each tetanus is composed of four pulses separated by 10 ms each (100 Hz). Hippocampal slices whose responses did not stabilize within a maximum period of 1 h or whose response increased or decreased disproportionately were discarded from the study and a new slice was analyzed. All records were filtered at 10 kHz and digitized at 5 kHz using Igor Pro software (WaveMetrics Inc., Lake Oswego, OR, USA).

### 2.4. Statistical Analysis

The results obtained were expressed as the mean ± standard error and represented through graphs. For the electrophysiology analysis, the U-Mann–Whitney test was applied as for the paired comparison, while the Kruskal–Wallis test was applied in the comparisons of more than two groups. In the case of the analysis of cognitive functions, an ANOVA test with repeated measures was applied. As a post hoc evaluation, Dunn’s test was used. A *p* value less than or equal to 0.05 was considered significant.

## 3. Results

### 3.1. TLTC Shows No Significant Changes in Recognition Memory in Alzheimer’s Disease Model Mice

As previously reported, the total distance traveled, and the time spent by the animal in the center of the arena are associated with anxiety [46]. During the habituation phase the anxious behavior of the groups (WT, WT + TLTC, ALZ, ALZ + TLTC) was assessed through the “Open Field Test” (OFT) [47]. The data indicate that there was no difference between the groups in both the total distance traveled (Figure 2C) and the number of corner entries (Figure 2B). Therefore, there is no evidence that TLTC treatment produces anxiety in mice. After OFT was performed, the object recognition memory study was conducted in order to analyze recall judgment and familiarity, processes involving the hippocampus and perirhinal cortex, respectively [48]. Tools used to assess recognition memory have become increasingly useful in basic and preclinical research, with the novel object recognition task (NOR) as the most commonly used [48]. The NOR task involves the memory of a familiar object in parallel with the detection and encoding of a novel object, i.e., mice will be more attracted to the novel object and will spend more time exploring it as compared to the familiar object. This preference for the novel object is considered as an indicator of the recognition of the familiar object [43]. The evaluation of the NOR was performed by measuring the percentage of novel object preference [44]. As expected, the WT group spent more time exploring the novel object compared to the ALZ group (Figure 2D). Furthermore, the ALZ group spent more time exploring the familiar object, which suggests an alteration in memory recognition, which is consistent with previous literature [49,50]. Regarding the groups treated with TLTC, it was observed that the WT + TLTC group exhibits similar behavior to the WT group (without treatment). However, the ALZ + TLTC group increased their preference for the novel object, although the results were not statistically significant.

### 3.2. TLTC Fully Rescues LTP and Partially Restores LTD in Alzheimer’s Disease Model Mice

Extensive evidence shows that learning induces changes at the synaptic level [48]. To study whether the effects of TLTC on memory recognition correlated with hippocampal synaptic efficacy, we next evaluated basal synaptic transmission and SP. To that end, we performed an input/output study and found that WT (black circles), ALZ (blue circles), and WT + TLTC (gray circles) groups showed similar increases in the fEPSP slope as stimulation increased, while only the ALZ + TLTC (green circles) group showed a significant increase compared with the WT + TLTC group (Figure 3). On the other hand, Figure 3D shows an aberrant increase in the ALZ group compared to the WT in the absence of phototherapy, which is consistent with previous studies [51]. Interestingly, phototherapy has a significant effect on mutant animals since TLTC normalized basal synaptic transmission in the ALZ + TLTC group reached levels compared to those found in the WT group (Figure 3C,D), without affecting the WT + TLTC group.

In addition, we evaluated the paired pulse facilitation (PPF), which is a type of short-term plasticity. PPF is achieved by applying two consecutive pulses separated by different intervals between the stimuli and that depend on the residual calcium released in the first pulse and this, in hippocampal neurons, facilitates the second pulse [52]. The PPF responses measured at hippocampal CA3-CA1 synapses of the different groups, are shown in Figure 3H. The application of TLTC did not modify in any sense the relationships of paired pulses at any of the time intervals to which the paired stimulus was applied. Similarly, we did not observe any appreciable difference between the responses for ALZ without light therapy compared to the WT group.

**Figure 4:**
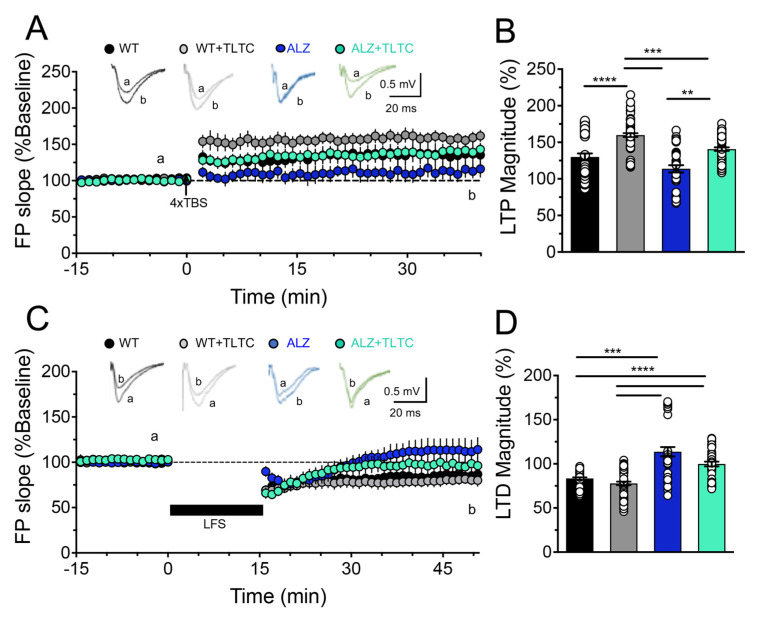
TLTC upgrades a Long-term potentiation (LTP) and Long-term depression (LTD) in the Schaffer-CA1 collateral pathway. (**A**) Insert, representative traces of field excitatory postsynaptic potentials (fEPSP) recorded 10 min before (a) and 40 min after (b) the application of the theta-burst stimulation protocol (The TBS are short trains of high-frequency of stimuli repeated at intervals of 200 ms). LTP induced by 4xTBS protocol at the Schaffer Collateral-CA1 synapse. The 4xTBS protocol was delivered during the time indicated by the black arrow. The dashed horizontal line indicates the baseline recording (before 4xTBS) as a 100% response. (**B**) Magnitude of average LTP measured in (b) for transgenic mice with Alzheimer’s disease without (ALZ, blue bar) and with TLTC (ALZ + TLTC, green bar), and control group, also subdivided into mice without (WT, black bar) and with TLTC (WT + TLTC, gray bar). (**C**) Insert, representative traces of field excitatory postsynaptic potentials (fEPSP) recorded 10 min before (a) and 50 min after (b) the application of the low-frequency stimulation protocol (LFS; 900 pulses/15 min). LTD) induced by LFS protocol at the Collaterals of Schaffer-CA1 synapses. The LFS protocol was delivered during the time indicated by the black bar. The dashed horizontal line indicates the baseline recording (before LFS) as a 100% response. (**D**) Magnitude of average LTD measured in (b) for transgenic mice with Alzheimer’s disease without (ALZ, blue bar) and with TLTC (ALZ + TLTC, green bar), and control group, also subdivided into mice without (WT, black bar) and with TLTC (WT + TLTC, gray bar). The number of asterisks indicate the strength of statistical significance (** *p* ≤ 0.01; *** *p* ≤ 0.001; **** *p* ≤ 0.0001).

To assess long-term plasticity, we used different protocols to induce LTP or LTD (Figure 4). Figure 4A,B shows the effects of the TLTC on LTP, where the WT mice without TLTC shows a robust and sustained LTP (black circles, WT = 150 ± 5.3%) after the application of the 4xTBS protocol similar to the same group with phototherapy (gray circles, WT + TLTC = 160.4 ± 5.3%). As expected, the LTP in the transgenic mice without TLTC is significantly diminished (blue circles, ALZ = 130.7 ± 5.9%). However, it can be observed that PBM reverses the LTP defect in transgenic mice, improving its induction and stability (green circles, ALZ + TLTC = 165.2 ± 4.2).

Finally, we evaluated the effect of TLTC on LTD. As observed in Figure 4C,D, the low-frequency protocol induced a decrease in synaptic efficacy in the WT mice with or without LED treatment. In both cases, the LTD was maintained for at least 40 min (black circles, WT = 90 ± 1.3%; and gray circles, WT + TLTC = 85.22 ± 1.9%). However, in the transgenic mice condition, the stimulation protocol leads to an induction of LTD that is not capable of being sustained over time and, furthermore, after fifteen minutes it becomes a potentiation that is sustained over time (blue circles, ALZ = 118.4 ± 4.8%). Interestingly, in the transgenic mice treated with TLTC, the aberrant potentiation induced by the LFS protocol was not observed. However, a transient LTD was induced but it was not maintained over time, reaching basal levels after 15 min (green circles, ALZ + TLTC = 104.4 ± 2.5%). This suggests that TLTC treatment partially ameliorated defects in LTD.

## 4. Discussion

Alzheimer’s disease (AD) is a neurodegenerative disorder whose exact pathophysiological mechanism remains unclear. However, there is increasing evidence suggesting synaptic dysfunction at the hippocampal level mediated by abnormal aggregation of Aβ proteins and Tau oligomers, leading to a decline in the individual’s cognitive functions [23]. Emerging non-invasive treatments such as PBM can be useful to minimize brain damage and therefore improve cognitive functions [53]. There are other non-invasive brain stimulation techniques demonstrating important advances in the cognitive neurorehabilitation of patients with AD, highlighting transcranial magnetic stimulation, transcranial direct current stimulation, transcutaneous nerve stimulation, and vagus nerve stimulation [54]. Interestingly, deep transcranial magnetic stimulation of the prefrontal cortex of AD patients improved attention, visuospatial, and executive functions [55], while repetitive transcranial magnetic stimulation of the dorsolateral prefrontal cortex may even regulate emotional processes by disrupting the reconsolidation of human fear memory [56]. These non-invasive magnetic brain stimulation techniques are also promising for the treatment of AD. However, these stimulation techniques may produce contraindications in patients with a history of epilepsy or in those taking stimulant medication. In this sense, it is necessary to apply safer brain stimulation technologies/techniques such as TLTC.

Regarding the mechanisms, it has been shown that TLTC can lead to cognitive benefits, improving memory and learning through the modulation of mitochondrial function, reducing inflammation, and helping the brain to repair itself by stimulating the neurogenesis process and increasing synaptogenesis in the cortex, hippocampus, and subventricular zone [57]. To date, the best described mechanism of action of phototherapy has been the reaction of cytochrome C oxidase (CCO), the main mitochondrial chromophore or photoacceptor molecule [58]. CCO plays a central role in eukaryotic cell bioenergetics by driving adenosine triphosphate (ATP) formation through oxidative phosphorylation, after transcranial PBM [59]. In addition, PBM can increase antiapoptotic proteins (Bcl2 and survivin), thus improving neuroprotection and neurorecovery [60]. Since AD has been linked to mitochondrial dysfunction, tPBM may be a viable treatment for AD [28]. One of the main results that emerge from this work is that transcranial PBM (TLTC) applied with a wavelength of 630 nm (TLTC), shows a tendency to improve cognitive abilities in a murine model of AD. It is likely that increasing the application time or the energy density of the therapy, in future experiments, a significant effect on behavior will be achieved, since our results are consistent with previously reported studies where PBM was able to reverse cognitive decline and improve memory [61]. In particular, recognition memory is one of the most affected during AD progression and our results using TLTC show a trend of improvement in transgenic mice where symptoms related to AD are manifested, without affecting other behavioral parameters, such as anxiety and locomotor activity.

These results are in agreement with those observed by Da Luz et al. (2017) [33] who observed, using PBM in mice with AD, a significant reduction in senile plaques together with a beneficial effect on behavioral and locomotor tasks. Moreover, Zhang et al. (2020) [56] demonstrated that PBM can effectively penetrate the brain, thereby reversing spatial learning/memory impairments and reducing senile plaque Aβ levels in AD model mice. In this sense, Cho et al. (2020) [57] suggested that the improvement in cognitive functions could be a consequence of the reduction of amyloid accumulation, neuronal loss, and gliosis. To explore the possible cellular mechanisms behind these improvements and given that memory induces changes at the neuronal level that correlates with changes in synaptic efficacy [62], we evaluated variations at the synaptic level in the animals that were treated with PBM. To the best of our knowledge, this is the first time that the effects of PBM on SP have been electrophysiologically described using a transgenic AD mouse model. Interestingly, we found that there was a noticeable and significant improvement on LTP in AD animals treated with PBM, reversing the defects at the synaptic level observed in this group, reaching basal levels. Furthermore, we found that TLTC partially reversed the aberrant LTD observed in the transgenic animals that develop AD (Figure 4). These results agree with Comerota et al. (2017) [51], who evaluated the effect of NIR treatment on synaptic vulnerability induced by Aβ oligomers using hippocampal slices from WT mice. Similarly, they found that NIR light therapy significantly restored LTP. To explore the effect of PBM on long-term plasticity, Shen et al. (2021) [58] showed that photobiomodulation suppresses JNK3 by activation of ERK/MKP7 to attenuate AMPA receptor endocytosis in Alzheimer’s disease, which has critical roles in LTP and LTD [59,60]. Furthermore, it is postulated that the emitted photons in the photobiomodulation (PBM) are absorbed by (CCO) mitochondrial to facilitate the availability of electrons for the reduction of molecular oxygen, increasing mitochondrial membrane potential, as well as the levels of (ATP) and cyclic adenosine monophosphate (cAMP) [61]. Moreover, the increase of ATP and cAMP leads to a dissociation of nitric oxide (NO), which in turn produces an enhancement of ATP and activates beneficial cellular pathways [28], overcoming mitochondrial dysfunctions observed in AD [62].

The results obtained in this work showed that transcranial light therapy (TLTC) can be an efficient alternative to enhance synaptic plasticity (SP) and a promising tool to repair cognitive properties impaired in AD patients. Although more in vivo trials with a larger number of patients are required, the development of devices that combine light stimulation with neuronal recording will lead to a more effective neuromodulation, especially with respect to cognitive, affective, and motor disorders [63]. An important aspect of this therapy is that it presents few adverse effects. In fact, in the work of Chao et al. (2019) [64] and Saltmarche et al. (2017) [35], the effects of PBM in vivo were evaluated in a group of patients with dementia, showing no adverse effects. These results differ from previous studies using pharmacological treatments where patients reported adverse symptoms such as vomiting, nausea or diarrhea [65,66].

The novelty of the present research relies on the scientific evidence provided on how transcranial light therapy exerts a positive effect on SP and possible benefit in cognitive functions in subjects with AD, using a specially designed light device for the experimental design. However, there are still future challenges to be faced, such as varying the exposure times of the treatment, as well as evaluating subjects with different degrees of cognitive impairment, while maintaining the wavelength of the light used.

## 5. Limitations and Future Directions

In our work, the adaptation of mice to the device was a critical step that limited the incorporation of stressed individuals into behavioral experiments. In this sense, we consider that a bigger sample would have resulted in higher statistical power and significance of our behavioral results. On the other hand, in most treatments that used PBM, various cellular effects are generated depending on the selected wavelength and time. Given that other studies showed that PBM at 630 nm generates a positive effect on the brain, we believed that a PBM for longer exposure times could emphasize the statistical differences in our behavioral results. Nevertheless, our results are promising and motivate us to carry out a larger study exploring the mechanisms of action of TLTC and its effects on AD pathological changes. Ultimately, we hope that these results will bring us closer to testing these revolutionary technologies on human AD patients with impaired cognitive abilities. To that end, it is necessary to explore the effects of PBM at the different stages of the AD continuum.

## 6. Conclusions

In general, the present study shows the beneficial effect of TLTC on neuronal plasticity in a murine model of AD, showing higher levels of LTP than those transgenic mice that were not exposed to the treatment, supporting the idea that TLTC may be a good alternative treatment for AD and associated synaptic deficit in humans. Our results also emphasize the importance of considering the use of non-pharmacological tools in AD, whose exposure times must be controlled, just as the therapeutic dose of a drug must be adjusted.

## Data Availability

Not applicable.
